# Cooperation and the evolution of intelligence

**DOI:** 10.1098/rspb.2012.0206

**Published:** 2012-04-11

**Authors:** Luke McNally, Sam P. Brown, Andrew L. Jackson

**Affiliations:** 1 Department of Zoology, School of Natural Sciences, Trinity College Dublin, Dublin 2, Republic of Ireland; 2 Centre for Biodiversity Research, Trinity College Dublin, Dublin 2, Republic of Ireland; 3 Centre for Immunity, Infection and Evolution, School of Biological Sciences, University of Edinburgh, Edinburgh EH9 3JT, UK

**Keywords:** reciprocity, Machiavellian intelligence, cognition, social brain, prisoner's dilemma, snowdrift game

## Abstract

The high levels of intelligence seen in humans, other primates, certain cetaceans and birds remain a major puzzle for evolutionary biologists, anthropologists and psychologists. It has long been held that social interactions provide the selection pressures necessary for the evolution of advanced cognitive abilities (the ‘social intelligence hypothesis’), and in recent years decision-making in the context of cooperative social interactions has been conjectured to be of particular importance. Here we use an artificial neural network model to show that selection for efficient decision-making in cooperative dilemmas can give rise to selection pressures for greater cognitive abilities, and that intelligent strategies can themselves select for greater intelligence, leading to a Machiavellian arms race. Our results provide mechanistic support for the social intelligence hypothesis, highlight the potential importance of cooperative behaviour in the evolution of intelligence and may help us to explain the distribution of cooperation with intelligence across taxa.

## 1. Introduction

Natural selection never favours excess; if a lower-cost solution is present, it is selected for. Intelligence is a hugely costly trait. The human brain is responsible for 25 per cent of total glucose use, 20 per cent of oxygen use and 15 per cent of our total cardiac output, although making up only 2 per cent of our total body weight [1]. Explaining the evolution of such a costly trait has been a long-standing goal in evolutionary biology, leading to a rich array of explanatory hypotheses, ranging from evasion of predators to intelligence acting as an adaptation for the evolution of culture [2–4]. Among the proposed explanations, arguably the most influential has been the ‘social intelligence hypothesis’, which posits that it is the varied demands of social interactions that have led to advanced intelligence [4–12].

In recent years, the cognitive demands of reciprocity, one of the mechanisms posited as important in the maintenance of cooperation in humans and other intelligent taxa, have been suggested to be a causal factor in the evolution of advanced intelligence and human language. This has been particularly apparent in the evolutionary game theory literature, where conjecture regarding this relationship is frequent [13–16]. Indeed, there is a rich history of work relating intelligence and reciprocity in the game theory literature, though most of this work has focused on the cognitive abilities required for the evolution of cooperation, rather than the possible role that the negotiation of these interactions has in the evolution of intelligence [17–24]. As well as the cognitive abilities required for the coordination of partners during cooperative acts, both direct (decisions based on what you do to me) and indirect (decisions based on what you do to others) reciprocity have additional demands in terms of the ability to remember previous interactions and to integrate across these interactions to make decisions in cooperative dilemmas [25–31]. These cognitive demands, combined with the occurrence of cooperative behaviour between unrelated individuals in intelligent taxa, suggest that selection for these mechanisms of cooperation could, at least in part, be responsible for advanced cognitive abilities [26].

The many subfields within the social intelligence hypothesis have shown a rich elaboration of verbal arguments, and data from comparative studies support many of their predictions [32–34]. However, verbal reasoning and comparative analysis alone are not sufficient to assess the relative merit of competing hypotheses [35]; mechanistic models are needed to assess the plausibility of these different explanations for advanced cognition.

Here, we use an artificial neural network model to focus on the potential for direct reciprocity, a behaviour that is widespread in humans, to select for advanced cognitive abilities. Rather than manufacturing some form of functional relationship between intelligence and fitness, we allow this relationship to emerge based on the demands of decision-making in two social dilemmas, and analyse the consequences for the evolution of intelligence.

## 2. Material and methods

### (a) The social dilemmas

In order to consider the dynamics of cooperative social interactions, we use the framework of two classic social dilemmas: the iterated prisoner's dilemma (IPD) and the iterated snowdrift game (ISD). In both games, two players must choose between cooperation and defection during repeated rounds. In the event of mutual cooperation or mutual defection, both players receive payoffs *R* or *P*, respectively, while a defector exploiting a cooperator gets *T* and the cooperator gets *S*. In the prisoner's dilemma, the benefit of an individual's cooperative behaviour goes to their opponent, while they pay all of the costs (e.g. food sharing, reciprocal coalitionary behaviour). This results in a payoff order of *T* > *R* > *P* > *S*. Here, the worst possible outcome for an individual is to cooperate while their opponent defects, while the best outcome is to defect while the opponent cooperates. In the snowdrift game, the benefits of cooperative behaviours are shared between opponents, and the costs are shared if both individuals cooperate (e.g. cooperative hunting, coalitionary behaviour with shared benefits). This results in a payoff order of *T* > *R* > *S* > *P*. Again, the best outcome for an individual is to defect while their opponent cooperates, though the worst possible outcome for an individual is for neither them nor their opponent to cooperate. In both games, the overall payoff (sum of both individual's payoffs) is greatest for mutual cooperation and lowest for mutual defection.

All of this means that the equilibrium frequency of cooperation for a single interaction (single-interaction Nash equilibrium) will be zero in the prisoner's dilemma but will be non-zero for a single-interaction snowdrift game [36]. These single-interaction Nash equilibria provide a useful benchmark against which to assess the effects of contingent behaviours (i.e. those that depend on the behaviour of others) in repeated interactions.

### (b) The neural network model

Any attempt to define a metric of intelligence will always be a contentious matter. However, comparative studies across taxa have usually focused on two main classes of brain properties as proxies of intelligence: metrics based on relative or absolute size of the brain or certain brain regions, and metrics based on more specific properties such as numbers of cortical neurons [37]. It is with this tradition in mind that we develop our artificial neural network model, with evolving network structure, using the number of neurons, *i*, as our proxy for intelligence. Each individual can display varying levels of intelligence, from simply being characterized by a binary response of always cooperate or always defect to large neural networks that possess complex neuronal structure, allowing for computations to inform decisions based on payoffs and the integration of longer-term memory into their current decision-making processes.

**Figure 1.**
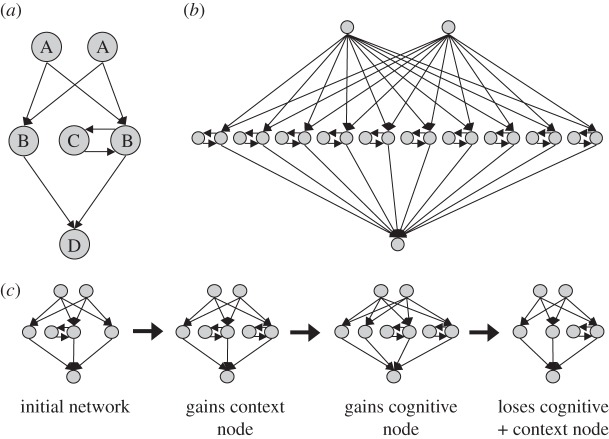
The artificial neural network model. (*a*) A schematic to aid in the understanding of our network structures is shown. Input nodes, which receive the payoffs of both players in the previous round, are labelled A. Cognitive nodes, which can receive input from both input and context nodes, are labelled B. Context nodes, which store the previous state of their cognitive node and return this state (times a weight) as input in the next round, are labelled C. The output node that receives inputs from the cognitive nodes and gives the individual's decision to cooperate or defect is labelled D. The most complex artificial neural network allowed in our simulations is shown in (*b*), possessing 10 cognitive nodes and 10 context nodes. A sample of a possible sequence of mutations to network structure is shown in (*c*). Individuals gain and lose cognitive and context nodes by random mutation. If a cognitive node with a connection to a context node is lost by mutation, the context node is also lost.

Each individual in our simulated populations possesses a neural network that determines their behaviour in social dilemmas (illustrated in figure 1). The networks each have two input nodes (which receive the payoffs of the individual and their opponent in the previous round as inputs) and one output node (giving the probability that they cooperate during their next interaction). The hidden layer of each individual's network has an evolving structure, possessing different numbers of cognitive and context nodes [38] (figure 1). Cognitive nodes allow for computation based on the values of network inputs and context nodes, which in turn allow for the build-up of memory based on previous states of their associated cognitive nodes.

Computation in the network is implemented via synchronous updating of nodes. The value of each input node is passed to each of the network's cognitive nodes, multiplied by the weight linking the two nodes. Each cognitive node is also passed the current value of their associated context node (if they possess one) multiplied by the weight linking the two nodes. The cognitive nodes sum across all of the weighted values that they receive and pass this value through a sigmoidal squashing function, resulting in a value between 0 and 1, analogous to a probability of activation. All context nodes are then passed the value of their associated cognitive nodes. This allows the context nodes to build up memory of previous interactions without having to store the actual sequence of events that have occurred. The internal states of these context nodes could be considered analogous to emotional states. Finally, the values at all cognitive nodes are then passed to the output node (multiplied by their weights), summed and again passed through a sigmoidal squashing function. This output gives the probability that the individual will cooperate in the current round. As the sigmoidal function asymptotes to 0 at –∞ and 1 at ∞, there will always be inherent noise in the network's probabilistic decision. This property of the function also means that it is easy to minimize noise in the network's behaviour if that behaviour shows a lack of contingency (as the node can always be near one of the asymptotes), while contingent behaviour will show greater noise (as switching is more difficult to achieve near the asymptotes). This formulation has intuitive appeal over simply adding extraneous noise to individual decisions, as in nature we would expect individuals to make few mistakes when their behaviour is non-contingent, while more complex decisions would be expected to be more error-prone. As the network cannot make decisions without an input, each individual has an additional trait encoding whether they cooperate or defect in the first round.

We allowed networks to evolve according to natural selection using a genetic algorithm where fitness is the mean payoff per round from the iterated games minus a penalty for the individual's intelligence, *i*. When individuals reproduce, mutations allow for the gain and loss of nodes from the hidden layer of their network with a fixed probability. Context nodes could only be gained if there was already a cognitive node present without an associated context node. The loss of a cognitive node with an associated node resulted also in the loss of the associated context node.

The addition of extra cognitive nodes gives networks the potential to perform complex computation based on payoffs by increasing the dimensions of internal representation of the network. The addition of context nodes gives the potential for the integration of longer-term memory of previous interactions in these computations. If an individual possessed no hidden layer nodes in its network, its behaviour in all rounds was decided by its first round move (i.e. they either always cooperated or always defected). The weights of each node in the network (arrowed lines in figure 1) and the threshold of each node (see the electronic supplementary material) were encoded as continuous genetic traits, again subject to mutation during reproduction. This means that, while the number of nodes in the network constrains the possible behavioural repertoire, it is the way that the constituent parts of the network interact that actually decides the individual's behaviour. In this way, our metric of intelligence assesses the potential for complex behaviour that the individual possesses, rather than the appropriateness or ‘wisdom’ of their behaviour, similarly to the measures of intelligence used in comparative studies.

### (c) Model implementation

In order to elucidate when selection favoured intelligence, we ran 10 replicates of our model for both the IPD and ISD, with each replicate lasting 50 000 generations. The payoff values used for all simulations were *R* = 6, *P* = 2, *T* = 7 and *S* = 1 for the IPD, and *R* = 5, *P* = 1, *T* = 8 and *S* = 2 for the ISD. The genetic algorithm was implemented as follows (see the electronic supplementary material for further details):

— an initial population of random networks was generated;
— each individual played every other individual in the population (50 individuals) in an IPD or ISD;
— each individual network's fitness was calculated as their mean payoff per round minus a fitness penalty for their level of intelligence, *i*;
— individuals were selected to reproduce asexually with probability proportional to their fitness;
— newly produced offspring underwent mutation of their network weights, node thresholds and network structure with constant probabilities;
— the previous generation died; and
— the algorithm returned to step 2 until 50 000 generations was reached.

During simulations we recorded the frequency of cooperation in the population, the intelligence of individuals (*i*) and assessments of the behaviour of individuals against a pre-determined test set of moves (see the electronic supplementary material). We then analysed the gradients of selection for intelligence across these simulations by taking selection for intelligence as the covariance between fitness and intelligence in any given generation [39]. As 50 million individual neural networks were simulated in our study, and individuals were not constrained to base their behaviour only on the previous move, our simulations generated a great diversity of strategies. In order to gain a coarse-grained overview of the strategic composition of the population, we clustered individuals based on their proximity to four canonical strategy types: always-defect-like, always-cooperate-like, tit-for-tat-like (do what your opponent did to you) and Pavlov-like (if your payoff is over a threshold, repeat your previous move). Assignment to each of these strategy types was based on which of these four strategies each individual network clustered closest to based on its behaviour against the test set. While this clustering is only a coarse-grained view, it allows assessment of the effects of shifts towards contingent cooperative strategies on selection for intelligence. Additionally, contingent human cooperation has previously clustered as either tit-for-tat-like or Pavlov-like [40], though longer-term memory is often included [41]. For full details of our data analysis, we direct readers to the electronic supplementary material.

## 3. Results

**Figure 2.**
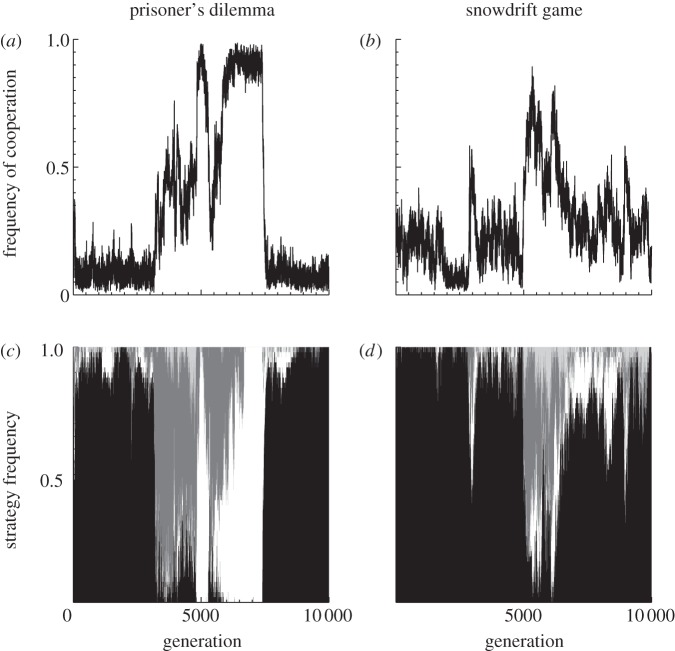
The emergence of intelligent strategies. Shown are the dynamics during 10 000 generation subsets of our simulations for the (*a,c*) prisoner's dilemma and the (*b,d*) snowdrift game. (*a*,*b*) Sample cycles in the frequency of cooperative acts in the population. (*b*,*d*) Frequencies of different strategy types (black, always-defect-like; white, always-cooperate-like; dark grey, tit-for-tat-like; light grey, Pavlov-like) as determined by clustering individuals with their nearest pure strategy (see §2 and electronic supplementary material for details). Transitions to cooperation are characterized by high numbers of contingent strategies, followed by the invasion of the always-cooperate strategy.

Our model shows the spontaneous evolutionary emergence of behaviours similar to strategies known to perform well in the IPD and ISD, such as tit-for-tat and Pavlov, as well as simple always-cooperate or always-defect strategies (figure 2) [42]. Although our networks’ behaviours are similar to these strategies, they often show integration over many previous rounds to decide on their next moves. For example, manual interrogation of networks revealed that, of the tit-for-tat type strategies that emerge, many are tit-for-2(or more)-tats, and many of the Pavlov-like strategies also show a threshold mechanism, switching to constant defection against opponents that show behaviour close to an always-defect strategy. Behaviour was observed that appeared to be close to many other strategies—for example, grim variants (cooperate until the opponent defects, then defect forever), though often requiring more than one defection to trigger permanent defection; false cooperator (cooperate first then switch to defection), though often giving another cooperative move after many defections; and many other variants of tit-for-tat such as 2-tits-for-1-tat and 2-tits-for-2-tats. It is worth noting that strategies of these types that use longer-term memory are observed in behavioural experiments of repeated games with noise [41]. These responsive strategies require greater cognitive abilities in order to carry out computations based on payoffs, memorize past rounds and integrate across them to make decisions, in comparison with the lower requirements of simply always cooperating or defecting. We hasten to add, however, that the strategies emerging only resemble these strategies; the strategies vary in a continuous manner and often incorporate memory over more rounds. Our goal here is not to describe the strategies that can emerge in repeated games, as there is already extensive literature on this topic (see table 2.1 in [43]), but rather to elucidate the potential effects of their evolution on selection for cognitive capacities.

**Figure 3.**
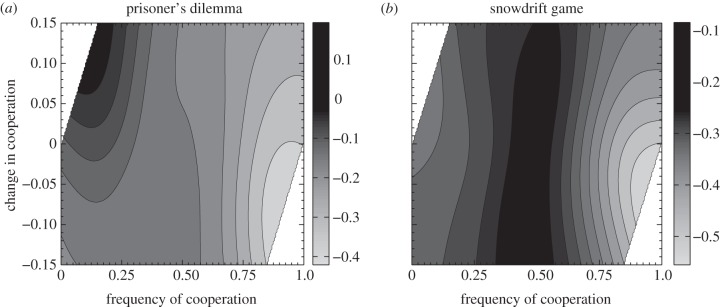
Selection for intelligence and transitions to cooperation. Shown is the level of selection for intelligence, taken as the covariance between fitness and intelligence, against the frequency of cooperation in the current generation and the change in the frequency of cooperation since the last generation. Darker shades indicate greater selection for intelligence. White areas indicate impossible value combinations. Selection for intelligence is maximized during transitions away from single-interaction equilibria, which are no cooperation for the IPD (*a*), and a frequency of cooperation of 0.23 for the ISD (*b*). Values displayed were smoothed using a Gaussian kernel.

In order to elucidate the causal factors leading to the evolution of more complex strategies, we analyse the gradient of selection for intelligence in response to population features such as the prevalence of cooperative acts. We find that the selection for intelligence is maximized as the level of cooperation in the population moves above the single-interaction Nash equilibria towards more cooperative regimes (figure 3). In the IPD, this maximum occurs during increases in cooperation from the single-interaction Nash equilibrium, whereas in the ISD selection for intelligence is maximized at levels of cooperation just above the single-interaction Nash equilibrium. This discrepancy between the games is explained by the different natures of their single-interaction Nash equilibria. In the IPD, this equilibrium is zero, meaning that declining cooperation near this equilibrium is caused by increases in the frequency of individuals that always defect, requiring only little cognitive ability. In the ISD, the equilibrium is non-zero (0.23 in our simulations), meaning that decreases in cooperation back towards the equilibrium can be caused by ‘meaner’ contingent strategies (e.g. 2-tits-for-1-tat and false-cooperator variants), as well as individuals that always defect. As a result, transitions back to the single-interaction Nash equilibrium in the ISD can in principle select for intelligence, while this is very unlikely in the IPD.

**Figure 4.**
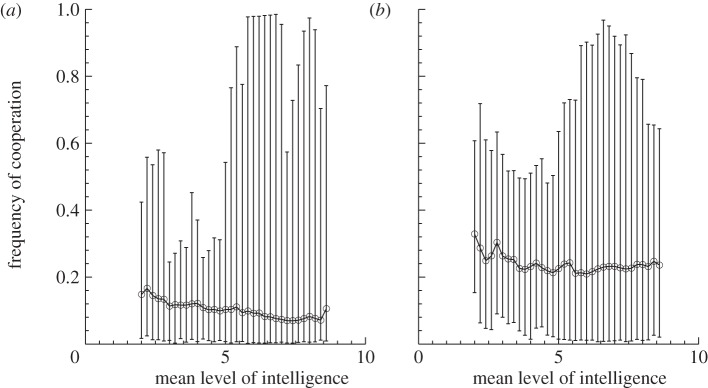
Intelligence and the distribution of cooperation. Shown are the relationships between the frequency of cooperative acts in the population and the mean level of intelligence in the population (*a*) for the prisoner's dilemma and (*b*) for the snowdrift game. Mean values are shown by the open circles and lines, and the ranges of the data are shown by the error bars. Greater intelligence decreases the mean level of cooperation in the IPD, while slightly increasing the mean level of cooperation in the ISD. The maximum and variance in the frequency of cooperative acts increases with intelligence, showing that the evolution of intelligent, contingent strategies leads to greater extremes in the frequency of cooperation.

We also find that increasing intelligence decreases the mean frequency of cooperative acts in the IPD (Spearman's rank correlation test; *ρ* = −0.2333, *p* < 0.001; figure 4*a*), while slightly increasing cooperation in the ISD (Spearmans' rank correlation test; *ρ* = 0.0089, *p* < 0.0001, figure 4*b*). Increasing intelligence increases the variance in the frequency of cooperative acts in the population in both the IPD (Breusch–Pagan test; intercept = 0.0294, slope = 0.0582, *p* < 0.0001; figure 4*a*) and the ISD (Breusch–Pagan test; intercept = 0.0309, slope = 0.0384, *p* < 0.001; figure 4*b*), showing that intelligence can facilitate greater extremes of cooperation. These results can be explained by assortment of individuals' cooperative acts [44]; the contingent strategies facilitated by increased intelligence allow an individual to increase the probability that they assort their cooperative acts with other cooperative acts. This leads to a synergistic process, where this increase in cooperation due to increased intelligence creates further opportunities for intelligent individuals to engage in mutual cooperation. However, as levels of cooperation increase further this feedback can break down, as there may be enough cooperation occurring for unconditional cooperators to increase in the population, allowing in turn for the invasion of unconditional defectors or ‘meaner’ intelligent strategies (e.g. grim variants, false-cooperator variants, etc.). This results in intelligence-facilitating cycles in the levels of cooperation seen in the population (figure 2), which increases both the variance in, and the maximum level of, cooperation.

**Figure 5.**
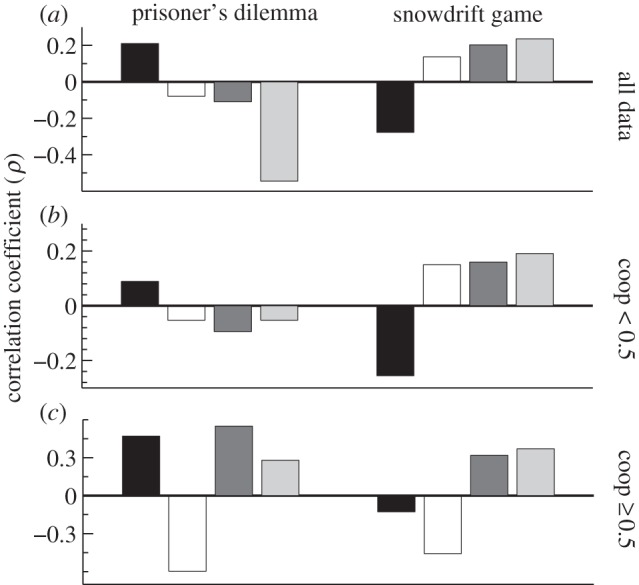
Strategic composition and selection for intelligence. The barplots show the Spearman rank correlation (*ρ*) between the frequency of each of our four strategy types and selection for intelligence for (*a*) all levels of cooperation, (*b*) low cooperation (frequency of cooperative acts less than 0.5) and (*c*) high cooperation (frequency of cooperative acts greater than or equal to 0.5). Tit-for-tat-like and Pavlov-like strategies always increase selection for intelligence in the ISD, but only when cooperation is high in the IPD. For further details, see electronic supplementary material, figure S1 and table S1.

In addition to dependency on the prevalence of, and change in, cooperative actions (figure 3), we find that intelligence can be subject to a Machiavellian runaway process [11,12]. In the ISD, as the frequency of contingent (intelligent) strategies increases, so too does selection for intelligence in the population (tit-for-tat-like strategies: Spearman's *ρ* = 0.2025, *p* < 0.0001; Pavlov-like strategies: *ρ* = 0.2352, *p* < 0.0001; see figure 5; electronic supplementary material, table S1 and figure S1). In the IPD, increasing frequencies of tit-for-tat and Pavlov reduces selection for intelligence at low levels of cooperation (frequency of cooperation <0.5; tit-for-tat: *ρ* = −0.0945, *p* < 0.0001; Pavlov: *ρ* = −0.0529, *p* < 0.0001), but does increase selection for intelligence when cooperation is more frequent (frequency of cooperation ≥ 0.5; tit-for-tat: *ρ* = 0.5491, *p* < 0.0001; Pavlov: *ρ* = 0.3187, *p* < 0.0001). The reason for this distinction between the IPD and the ISD is that there must already be some cooperation occurring for cooperative contingent strategies to be favoured via their ability to assort cooperative acts. In the ISD, the partially cooperative single-interaction equilibrium provides sufficient baseline cooperation for tit-for-tat and Pavlov to be favoured, whereas in the IPD the single-interaction equilibrium of zero cooperation means that unless contingent strategies (or random drift) have already increased cooperation, tit-for-tat-like and Pavlov-like strategies cannot be favoured, and hence cannot lead to an arms race. Note that it is still the case that intelligence is selected for in the IPD when cooperative acts are rare yet increasing (figure 3*a*). However, the cooperative acts that drive selection for intelligence in this case are generated by less cooperative contingent strategies, which cluster with always-defect as their closest pure strategy (*ρ* = 0.1095, *p* < 0.0001; see electronic supplementary material, table S1 and figure S1). This means that there is a succession in the arms race in the IPD, with ‘mean’ contingent strategies initially increasing selection for intelligence at low cooperation, and ‘kind’ contingent strategies increasing selection for intelligence as cooperation increases.

It is not any particular single strategy that drives these arms races; rather, as the complexity of the strategies in the population increases, there is selection for other complex strategies to outwit them. Unlike previous analyses where fixed strategies or strategies with constrained memory were used, our open-ended system allows for near infinite strategic variations to outwit opponents. In this way, selection for intelligence occurs owing to a constantly shifting strategic environment, where the ‘best response’ to the population of strategies can be shifting from generation to generation. Increases in memory allow for the potential of the recognition of opponents’ strategies, allowing for the alteration of one's own strategy in response (e.g. Pavlov-like strategies that can recognize individuals that always defect). In turn this can allow for attempts to deceive opponents regarding one's own strategy (e.g. false cooperators).

## 4. Discussion

It is important here to note the closed nature of our model system; individuals can only choose within one social task (whether to cooperate or defect against another individual based on their behaviour in previous interactions with them). However, our results may apply in principle to other social scenarios where individuals use strategies to decide who to cooperate with or when to cooperate, such as indirect reciprocity [17,18], policing/punishment [45,46] and partner choice [47–49]. Along with kin selection, these are the major mechanisms thought to lead to transitions to cooperative groups. As the intelligence of an individual increases, it is likely that more of these behavioural repertoires will become available to them, with increased intelligence acting as a pre-adaptation. For example, increased intelligence owing to selection for direct reciprocity could facilitate the evolution of indirect reciprocity or partner choice, highlighting the contingent nature of social evolution in multiple strategic dimensions [50]. The facilitation of new social behaviours due to emergent intelligence could allow for a perpetuated Machiavellian arms race leading to ever-greater levels of intelligence. Additionally, in our simulations populations evolved to play only a single game (either the IPD or ISD). The simultaneous play of multiple games could greatly increase strategic complexity, with the possibility of the integration of information from previous interactions in games with different payoff structures into the decision-making process.

It has previously been suggested that the pinnacles of cooperative behaviour in nature show a bimodal distribution with intelligence, with the most cooperative species displaying either limited cognition (e.g. microbes, social hymenoptera) or exceptional intelligence (e.g. humans and other primates, certain cetaceans and birds) [26]. It is clear in the former case that cooperation has evolved primarily owing to combinations of kin selection and ecological factors [51]. However, in the latter case, kin selection is not the only mechanism leading to cooperation, and may not even be the most important. A recent study has in fact suggested that relatedness was too low in human hunter–gatherer groups for kin selection to drive the evolution of human cooperation [52]. In highly intelligent species, contingent behaviours (reciprocity, partner choice, etc.) appear to have been important in the evolution of cooperation [26]. Our results may help us to explain this pattern by showing that the selection for appropriate behavioural assortment of cooperative acts can lead to selection for greater cognitive abilities and Machiavellian arms races, and that intelligence facilitates greater extremes of cooperation. Additionally, although kin selection is still of importance in highly intelligent taxa, high relatedness may hinder the evolution of intelligence by driving unconditional cooperation to fixation in the population, without any need of contingent behaviours.

A trait as complex as advanced intelligence is likely to have evolved owing to a combination of several factors rather than a single factor [4]. Along with the social intelligence hypothesis, many other theories attempting to explain the evolution of advanced intelligence have been suggested, among them that intelligence is an adaptation for tool use [53,54], that intelligence is an adaptation for social learning and the accumulation of culture [55–57], and that intelligence is the result of sexual selection [58]. All of these theories are supported by evidence from at least some of the most intelligent animals. However, the difficulty lies in disentangling the traits that are causal factors in the evolution of intelligence from those that are by-products of advanced intelligence. The combination of game theoretic frameworks and artificial neural network models presented here may provide a framework for the evaluation of the mechanistic strengths of these different hypotheses. While previous models have sought to relate cooperation and intelligence, the focus has most frequently been on the cognitive requirements of cooperation, rather than on the selection for intelligence. Many of these models have lacked an explicit brain structure [17–22], and among those studies that have used artificial neural networks, we know of no examples where the network structure was allowed to freely evolve or implications of selection for decision-making strategies for the evolution of intelligence were directly addressed [23,24]. While artificial neural networks are not real brains, relying on abstraction of the activity of millions of real neurons down to a manageable number of artificial neurons, they can provide insight into the dynamics of cognitive evolution and allow for the flexible evolution of behaviour [59]. Our results show that, in a freely evolving system, selection for efficient decision-making in social interactions can give rise to selection pressures for advanced cognition, supporting the view that the transition to the cooperative groups seen in the most intelligent taxa may be the key to their intellect.
